# L1CAM further stratifies endometrial carcinoma patients with no specific molecular risk profile

**DOI:** 10.1038/s41416-018-0187-6

**Published:** 2018-07-27

**Authors:** Felix KF Kommoss, Anthony N. Karnezis, Friedrich Kommoss, Aline Talhouk, Florin-Andrei Taran, Annette Staebler, C. Blake Gilks, David G. Huntsman, Bernhard Krämer, Sara Y. Brucker, Jessica N. McAlpine, Stefan Kommoss

**Affiliations:** 1 Institute of Pathology, Heidelberg University Hospital, Heidelberg, Germany; 2 Department of Pathology and Laboratory Medicine, University of British Columbia and British Columbia Cancer Agency, Vancouver, BC Canada; 3 Institute of Pathology, Im Medizin Campus Bodensee, Friedrichshafen, Germany; 4 Department of Women’s Health, Tübingen University Hospital, Tübingen, Germany; 5 Institute of Pathology, University Hospital Tübingen, Tübingen, Germany; 6 Department of Pathology and Laboratory Medicine, University of British Columbia and Vancouver General Hospital, Vancouver, BC Canada; 7 University of British Columbia, Department of Gynecology and Obstetrics, Division of Gynecologic Oncology, Vancouver, BC Canada

**Keywords:** Endometrial cancer, Risk factors, Biomarkers, Molecular medicine, Outcomes research

## Abstract

**Background:**

The newly developed Proactive Molecular Risk Classifier for Endometrial Cancer (ProMisE) has consistently been shown to be prognostically significant in endometrial carcinomas (EC). Recently, we and others have demonstrated L1 cell-adhesion molecule (L1CAM) to be a significant indicator of high-risk disease in EC. In the current study, it was our aim to determine the prognostic significance of aberrant L1CAM expression in ProMisE subgroups in a large, single centre, population-based EC cohort.

**Methods:**

ProMisE (POLE; MMR-D; p53 wt/NSMP; p53 abn) classification results from a cohort of 452 EC were available for analysis. L1CAM expression was studied by immunohistochemistry on whole slides. Correlations between clinicopathological data and survival were calculated.

**Results:**

Expression of L1CAM was most frequent in p53 abnormal tumours (80%). L1CAM status was predictive of worse outcome among tumours with no specific molecular profile (p53 wt/NSMP) (*p* < 0.0001). Among p53 wt/NSMP EC, L1CAM remained a significant prognosticator for disease-specific survival after multivariate analysis (*p* = 0.035).

**Conclusion:**

L1CAM status was able to significantly stratify risk among tumours of the large p53 wt/NSMP ProMisE subgroup of EC. Furthermore, our study confirms a highly significant correlation between mutation-type p53 immunostaining and abnormal L1CAM expression in EC.

## Background

Endometrial cancer (EC) is the most common gynecological malignancy in women in the western world.^1^ EC patient treatment is currently based on risk classification, for which clinicopathological criteria, such as histological type, FIGO grade and stage are key parameters.^2–5^ However, several studies have shown the evaluation of pathological features such as grade and histotype to be of only limited reproducibility, particularly in high-grade tumours.^6–8^ Over the past years, molecular studies have looked into identifying more reproducible prognosticators in EC with the aim of creating a better risk classification system.

An important step has been the genomic characterisation of EC by The Cancer Genome Atlas (TCGA) group and others, defining four prognostically distinct molecular subgroups.^9,10^ In order to simplify the methodologies and to lower cost involved in identifying these molecular subgroups, research teams have recently described surrogate markers that can be studied more easily.^11–15^ Talhouk et al. have developed the Proactive Molecular Risk Classifier for Endometrial Cancer (ProMisE), demonstrating an easy pragmatic classification system for ECs.^11,12,15^ The four prognostic ProMisE subgroups are as follows: DNA Polymerase epsilon exonuclease domain mutation (POLE), mismatch repair deficiency (MMR-D), p53 wild-type (p53 wt) and p53 abnormal (p53 abn).^11^ Tumours within the p53 wt group, which do not harbour mutations of neither POLE, mismatch repair genes nor abnormal p53, are also referred to as tumours with “No Specific Molecular Profile, NSMP”.^16^

The L1 neuronal cell-adhesion molecule (L1CAM/CD171) has recently gained attention as a specific prognosticator and potential therapeutic target in EC and other tumours.^17^ Multiple studies have shown the prognostic significance of L1CAM immunohistochemistry (IHC) in large cohorts of EC.^18–23^ The upregulation of L1CAM was found to be a major driver for tumour cell motility and to be closely associated with the process of epithelial to mesenchymal transition (EMT).^24–28^ Tumours showing histological evidence of EMT are frequently biologically aggressive neoplasms and tend to present at an advanced tumour stage. The downregulation of E-Cadherin and hormonal receptors, and the upregulation of L1CAM have been described as main features of EMT in EC.^29,30^ Furthermore, multiple studies depict a connection between L1CAM expression and type II EC histology (serous/clear-cell histotype), known to display high rates of abnormal p53 status.^18,19,31^ So far, specific regulations of L1CAM expression on a transcriptional level have remained enigmatic. Recent reports have suggested a strong association between L1CAM expression and mutant-type p53 immunoreactivity; however, there is also evidence suggesting a p53-independent mechanism of L1CAM expression in multiple molecular subgroups.^31,32^

Recently, Karnezis et al. correlated L1CAM and other immunohistochemical markers with ProMisE subgroups in a population-based cohort of EC and found high L1CAM expression to be highly associated with tumours showing mutation-type p53 expression and to identify poor prognosis tumours within MMR-D and p53 wt molecular subgroups.^31^ Stelloo et al. found similar results in a large, non-population-based cohort of EC patients (PORTEC).^14^

The aim of our study was to investigate the correlation between ProMisE subgroups and pathological L1CAM expression in a large, single centre, population-based cohort. Specifically, we wanted to study the potential of L1CAM to improve molecular EC classification, especially within the intermediate-risk molecular subgroups (MMR-D and p53wt/NSMP). We discuss possible mechanisms by which L1CAM expression may lead to aggressive clinical behaviour in EC.

## Methods

### Study cohort

Patients treated for primary EC at the University Hospital Tübingen between 2003 and 2013 were identified and clinical data, tissue samples and specialised gynecopathological review data were collected. Risk classification was performed applying current ESMO 2016 criteria.^2^ Patients with synchronous or metachronous second malignancies were excluded. Follow-up data was obtained from the Tübingen University Hospital Clinical Cancer Registry and subsequently updated, allowing for evaluation of overall survival (OS) and disease-specific survival (DSS). The Tübingen University Independent Ethics Committee issued study approval.

### Molecular classification

The ProMisE classification system applied in this study has previously been described in detail.^15^ Briefly, tumours were evaluated for 4 prognostic subgroups: Polymerase epsilon exonuclease domain mutations (POLE) detected through sequencing (exon 9–14), mismatch repair deficiency (MMR-D) evaluated using IHC for the absence of MSH6 or PMS2, and *TP53* mutation evaluated using IHC for p53, revealing p53 wild-type (p53 wt/NSMP) and p53 abnormal (p53 abn) subgroups.

### Immunohistochemistry

L1CAM IHC (Clone 14.10, Covance; 1:50 dilution) was performed according to established protocols.^19^ L1CAM expression was scored according to percentage of positivity in tumour cells (score 0 = 0%, score 1 = 1–10%, score 2 = > 10–50% and score 3 = > 50%; Supplementary Figure 1) and tumours were determined L1CAM positive, if ≥10% (score 2 and 3) of epithelial tumour cells showed membranous L1CAM staining. The threshold for L1CAM positivity (≥10% of tumour cells) was previously established by Zeimet et al., based on the cutoff that best correlated with prognosis.^19^ Evaluation of L1CAM IHC results was performed independently by two of the authors (FKFK, AS), who were blinded for outcome and ProMisE subgroups. In all cases with discrepant scoring results, a consensus was reached between both investigators.

### Statistical analysis

Associations between L1CAM, ProMisE subgroups and other clinicopathological parameters were assessed using the fisher’s-exact-test (FET). Survival analysis for OS and DSS were calculated using the Kaplan–Meier and cox-proportional hazard model. P-values (Likelihood-ratio test, LRT) and corresponding confidence intervals (CI) of 95% were recorded.

## Results

### Study cohort and molecular classification

Four hundred fifty-two cases of primary EC were available for the current study. Patient follow-up data allowed for OS calculation in all 452 cases, and for DSS in 450 cases. Median follow-up time was 68 months (1–158 months). Study cohort characteristics, including ProMisE classification are given in Table 1 and are derived from previously published data.^15^ A small proportion of tumours (8 cases, 1.8%) demonstrated more than one molecular feature (e.g. POLE and p53 abn, or POLE and MMR-D), and were classified as POLE or MMR-D, respectively, by strictly following the ProMisE decision tree, which dictates the order in which tumours are assigned to a specific molecular subgroup.^15^

**Table 1:** Clinicopathological data and L1CAM

	Total	L1CAM negative	L1CAM positive	*P* value
Number of patients	452 (100%)	355 (78.5%)	97 (21.5%)	
Clinicopathological parameters				
Age at diagnosis (yrs)				
Mean (±sd)	60.2 (±11.5)	63.8 (±11.6)	69.3 (±10.1)	<0.001
Median	65.3	64.1	69.8	
BMI				
Mean (±sd)	29 (±7.7)	29.4 (±8)	27.5 (±6.1)	0.02
Median	27.7	28	26.8	
Missing	20	16	4	
ProMisE classification				
POLE	42 (9.3%)	35 (9.8%)	7 (7.2%)	<0.001
MMR-D	127 (28.1%)	101 (28.5%)	26 (26.8%)	
p53 wt/NSMP	228 (50.4%)	209 (58.9%)	19 (19.6%)	
p53 abn	55 (12.2%)	10 (2.8%)	45 (46.4%)	
Stage (FIGO 2009)				
I	365 (80.8%)	303 (85.3%)	62 (63.9%)	<0.001
II–IV	87 (19.2%)	52 (14.7%)	35 (36.1%)	
Tumour grade				
Grade 1	282 (62.4%)	267 (75.2%)	15 (15.5%)	<0.001
Grade 2	75 (16.6%)	58 (16.3%)	17 (17.5%)	
Grade 3	95 (21%)	30 (8.5%)	65 (67%)	
Histology				
Endometrioid	397 (87.8%)	349 (98.3%)	48 (49.5%)	<0.001
Non-endometrioid	55 (12.2%)	6 (1.7%)	49 (50.5%)	
LVSI				
Negative	388 (85.8%)	315 (88.7%)	73 (75.3%)	0.005
Positive	60 (13.3%)	38 (10.7%)	22 (22.7%)	
Missing	4 (0.9%)	2 (0.6%)	2 (2%)	
Adjuvant treatment				
None	171 (37.8%)	151 (42.5%)	20 (20.6%)	<0.001
Any	281 (62.2%)	204 (57.5%)	77 (79.4%)	
ESMO risk classification 2016				
Low	230 (50.9%)	213 (60%)	17 (17.5%)	<0.001
Intermediate	64 (14.1%)	58 (16.4%)	6 (6.2%)	
High-intermediate	27 (6%)	22 (6.2%)	5 (5.2%)	
High	131 (29%)	62 (17.4%)	69 (71.1%)	

### L1CAM Immunohistochemistry

L1CAM IHC was performed in all 452 cases, of which results for ESMO non-high-risk tumours were previously reported.^23^ L1CAM was completely negative (score 0) in 320/452 (70.8%) tumours, showed minimal staining (score 1, 1–10% of tumour cells) in 35/452 (7.7%), and positive staining ( ≥ 10% of tumour cells) in 97/452 (21.5%) tumours. Of positive tumours, 59/452 (13.1%) showed moderate L1CAM staining (score 2, > 10–50%), and 38/452 (8.4%) showed strong L1CAM expression (score 3, > 50%).

### L1CAM and ProMisE molecular subgroups

**Fig. 1:**
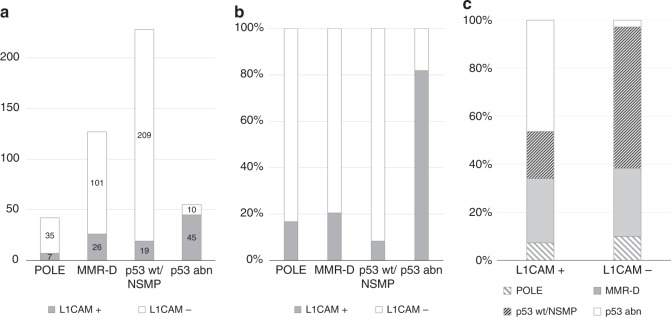
L1CAM-positive cases among ProMisE subgroups: **a** Represented as absolute numbers **b** Represented as the percentage of each molecular subgroup. **c** Percentage of ProMisE subgroups among L1CAM positive and negative EC

Figure 1 illustrates L1CAM positivity rates within ProMisE subgroups. 7/42 (16.7%) of tumours with POLE, 26/127 (20.5 %) of tumours with MMR-D, 19/228 (8.3%) of tumours with p53 wt/NSMP and 45/55 (81.8%) of tumours with p53 abn were L1CAM positive (Table 1; Fig. 1a, b). Inversely, ProMisE subgroups were distributed among L1CAM-positive tumours as follows: 7/97 (7.2%) POLE, 26/97 (26.8%) MMR-D, 19/97 (19.7%) p53 wt/NSMP, and 45/97 (46.3%) p53 abn (Fig. 1c). The differences in percentage of L1CAM positivity among ProMisE subgroups was statistically significant (*p* < 0.001).

### Survival analysis of the whole cohort

**Fig. 2:**
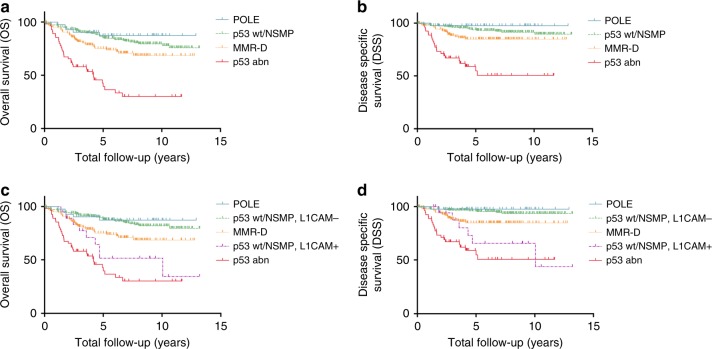
Kaplan–Meier survival analysis considering ProMisE subgroups and L1CAM expression. OS and DSS of ProMisE subgroups before (**a**, **b**) and after (**c** and **d**) stratifying for L1CAM status within the p53 wt/NSMP subgroup. **a** Five-year OS rates of 87.3% for POLE tumours, 85.2% for p53 wt/NSMP tumours, 75.0% for MMR-D tumours, and 39.6% for p53 abn tumours (*p* < 0.001). **b** Five-year DSS rates of 97.6% for POLE tumours, 93.6% for p53 wt/NSMP tumours, 84.7% for MMR-D tumours, and 54.8% for p53 abn tumours (*p* < 0.001) **c** Five-year OS rates of 87.3% for POLE tumours, 88.2% for p53 wt/NSMP, L1CAM negative tumours, 75.0% for MMR-D tumours, 51.5% for p53 wt/NSMP, L1CAM-positive tumours and 39.6% for p53 abn tumours (*p* < 0.001) **d** Five-year DSS rates of 97.6% for POLE tumours, 95.5% for p53 wt/NSMP, L1CAM negative tumours, 84.7% for MMR-D tumours, 65.5% for p53 wt/NSMP, L1CAM-positive tumours and 54.8% for p53 abn tumours for disease-specific 5-year survival (*p* < 0.001)

Univariate survival analyses of L1CAM within each ProMisE subgroup (Table 2A) showed that L1CAM status had a statistically significant prognostic impact only among p53 wt/NSMP tumours. Here, L1CAM positivity was accompanied with a Hazard Ratio (HR) of 3.78 (CI 1.69–7.61; *p* = 0.002) for OS and 7.82 (CI 2.65–21.12; *p* = 0.0008) for DSS (Table 2A). Kaplan–Meier analyses showed significantly worse outcome for L1CAM positive, p53 wt/NSMP tumours with 5-year OS rates of 88.2% for L1CAM negative and 51.5% for L1CAM-positive tumours (Fig. 2c; *p* < 0.001), and 95.5% for L1CAM negative and 65.5% for L1CAM-positive tumours for 5-year DSS (Fig. 2d; *p* < 0.001). For the remaining three subgroups, only a prognostic trend for L1CAM positivity and worse outcome was observed.

**Table 2:** **A** Univariate survival analysis (OS and DSS) considering L1CAM among ProMisE subgroups. **B** Multivariate survival analysis (OS and DSS) considering L1CAM, age and ProMisE subgroups

		OS			DSS	
	# of events / *n*	Hazard ratio (95% CI)	LRT *P* value	# of events / n	Hazard ratio (95% CI)	LRT *P* value
A						
POLE						
L1CAM negative (ref)	5/42	—		1/42	—	
L1CAM positive		1.39 (0.07–9.45)	0.7		NA	0.06
P53 wt/NSMP						
L1CAM negative (ref)	41/228	—		16/227	—	
L1CAM positive		3.78 (1.69–7.61)	0.002		7.82 (2.65–21.12)	0.001
MMR-D						
L1CAM negative (ref)	33/127	—		16/126	—	
L1CAM positive		1.25 (0.53–2.66)	0.6		1.26 (0.35–3.63)	0.7
P53 abn						
L1CAM negative (ref)	33/55	—		22/55	—	
L1CAM positive		0.87 (0.39–2.17)	0.7		1.06 (0.40–3.68)	0.9
B						
Age	112 / 452			55 / 450		
		34.16 (10.16–117)	<0.001		4.61 (0.83–25.67)	0.08
ProMisE	112 / 452			55 / 450		
p53 wt/NSMP (ref)		—			—	
POLE		0.84 (0.28–1.95)	<0.001		0.32 (0.02–1.61)	0.002
MMR-D		1.49 (0.93–2.38)			1.73 (0.86–3.46)	
p53 abn		3.18 (1.71–5.97)			3.97 (1.74–9.31)	
L1CAM status	112 / 452			55 / 450		
Negative (ref)		—			—	
Positive		1.33 (0.77–2.22)	0.3		2.05 (1.00–4.10)	0.05

The p53 wt/NSMP subgroup was further stratified using L1CAM status in a univariate model (Table 3). Here the L1CAM-positive subgroup of p53 wt/NSMP tumours was at higher risk for fatal outcome, when compared to the p53 wt/NSMP, L1CAM negative subgroup with a HR of 6.94 (CI 2.56–18.74; *p* < *0.001*) for DSS. In the same statistical model p53 abn tumours where also at higher risk for disease-specific death with a HR of 11.52 (CI 5.55–23.90; *p* < *0.001*).

**Table 3:** Univariate survival analysis (OS and DSS) considering ProMisE subgroups and L1CAM status within the p53 wt/NSMP subgroup

		OS			DSS	
	# of events / *n*	Hazard ratio (95% CI)	LRT *P* value	# of events / *n*	Hazard ratio (95% CI)	LRT *P* value
ProMisE	112 / 452			55 / 450		
p53 wt/NSMP L1CAM- (ref)		—			—	
POLE		0.81 (0.32–2.08)	<0.001		0.46 (0.06–3.58)	<0.001
MMR-D		2.07 (1.27–3.37)			2.99 (1.40–6.40)	
p53 wt/NSMP L1CAM+		3.70 (1.77–7.76)			6.94 (2.56–18.74)	
p53 abn		6.38 (3.90–10.42)			11.52 (5.55–23.90)	

In a multivariate analysis including ProMisE subgroups, age and L1CAM status (Table 2B), L1CAM status showed a trend toward prognostically significance for DSS; ProMisE subgroups were prognostically significant for OS and DSS. HR for L1CAM-positive tumours was 1.33 (CI 0.77–2.22; *p* = 0.3) for OS and 2.05 (CI: 1.00–4.10; *p* = 0.05) for DSS. There were relatively few L1CAM-positive tumours, which limited our power to study additional variables in the model using all four ProMisE subgroups.

### p53 wt/NSMP subgroup analyses

Detailed statistical subgroup analyses were performed on the 228 p53 wt/NSMP tumours, which are summarised in Table 4 and Supplementary Table 1. Among p53 wt/NSMP tumours L1CAM positivity was significantly associated with high tumour grade (FIGO grade 3) and high FIGO stage (II–IV) (Supplementary Table 1). In a multivariate model only including factors available from preoperative biopsy or curettage samples (age, histotype, and FIGO grade), L1CAM status was a strong and independent prognosticator for DSS within the p53 wt/NSMP subgroup. HR for L1CAM positivity was 2.43 (0.99–5.41; *p* = 0.052) for OS and 3.80 (CI: 1.10–12.16; *p* = 0.035) for DSS (Table 4A). In a multivariate model including factors available from hysterectomy specimens [age, histotype, FIGO grade, FIGO stage, and lymphovascular space invasion (LVSI)], L1CAM status remained prognostic for DSS (HR of 4.03, *p* = 0.035, Table 4B).

**Table 4:** Multivariate survival analysis (OS and DSS) in the p53 wt/NSMP ProMisE subgroup. L1CAM and established clinicopathological risk factors available (A) preoperatively and (B) postoperatively

		OS			DSS	
	# of events / *n*	Hazard ratio (95% CI)	LRT *P* value	# of events / *n*	Hazard ratio (95% CI)	LRT *P* value
A						
Age	41/228			16/227		
		1.05 (1.03–1.08)	<0.001		1.03 (0.98–1.08)	0.2
L1CAM	41/228			16/227		
Negative (ref)		—			—	
Positive		2.43 (0.99–5.41)	0.052		3.80 (1.10–12.16)	0.035
Tumour grade	41/228			16/227		
Grade 1 and 2 (ref)		—			—	
Grade 3		2.70 (1.00–6.40)	0.049		4.97 (1.33–16.55)	0.019
Histology	41/228			16/227		
Endometrioid (ref)		—			—	
Non-endometrioid		1.29 (0.07–7.45)	0.8		1.54 (0.08–10.00)	0.7
B						
Age	41/228			16/227		
		1.05 (1.02–1.08)	<0.001		1.04 (0.99–1.09)	0.068
L1CAM	41/228			16/227		
Negative (ref)		—			—	
Positive		2.26 (0.88–9.29)	0.08		4.03 (1.11–13.74)	0.035
Tumour grade	41/228			16/227		
Grade 1 and 2 (ref)		—			—	
Grade 3		1.84 (0.65–4.62)	0.2		2.73 (0.75–8.85)	0.1
Histology	41/228			16/227		
Endometrioid (ref)		—			—	
Non-endometrioid		1.56 (0.08–9.29)	0.7		3.43 (0.17–24.53)	0.3
LVSI	41/228			16/227		
Negative (ref)		—			—	
positive		2.64 (0.98–6.02)	0.2		9.65 (2.99–30.00)	<0.001
Stage (FIGO 2009)	41/228			16/227		
Stage I (ref)		—			—	
Stage II–IV		2.20 (1.04–4.46)	0.04		3.42 (1.17–10.33)	0.024

## Discussion

The recent introduction of clinically practical molecular classification systems of EC such as ProMisE is an important advancement in EC tumour classification by focusing on molecular parameters that have better reproducibility compared to the current WHO system, which is solely based on histomorphology.^11,12,15^ However, in order to guide surgery and/or adjuvant treatment there is still a need to further refine molecular risk stratification. This is most important for intermediate-risk groups of MMR-D and p53 wt/NSMP tumours, which constitute a large percentage of cases (64–79%).^11,12,15^ Our study demonstrates that L1CAM expression correlates with high grade, high stage, and poor prognosis for OS and DSS within the p53 wt/NSMP subgroup of EC, which agrees with previous studies on similar cohorts.^14,31^

The fact that L1CAM expression in our study was prognostic in the intermediate-risk group of p53 wt/NSMP tumours using both preoperative and postoperative variables indicates that the addition of L1CAM IHC to the ProMisE (or similar) molecular classifier could add useful information for both prognosis and treatment (Table 4). The expression of L1CAM in p53 wt/NSMP endometrioid carcinoma on curettage specimens might indicate the need to perform more aggressive surgery due to the correlation of L1CAM with advanced stage, similar to p53 abn molecular subgroup tumours, which commonly overexpress L1CAM (Table 1). After hysterectomy, patients with p53 wt/NSMP, L1CAM-positive tumours were at similar risk for fatal outcome when compared to patients with p53 abn tumours (Table 3 and Fig. 2). These patients might benefit from the addition of adjuvant therapy, even if current ESMO criteria indicate a low-risk situation. Similarly, molecular testing is already well established to determine the need for adjuvant treatment in early-stage breast cancer patients.^33,34^

A similar negative prognostic effect of L1CAM expression in the other intermediate-risk group of MMR-D tumours was recently demonstrated;^14,31^ we observed a similar trend; however, statistical significance was not reached in our cohort (Table 2A); our study cohort is heterogeneous in stage, ESMO-risk group, treatment, and has relatively few L1CAM-positive tumours, which limited our power to study the prognostic significance of L1CAM in specific subgroups of tumours. If their finding is reproduced in other studies, it may further expand the potential of L1CAM IHC to refine the prognosis of intermediate-risk tumours.

While L1CAM-positive tumours clearly have worse outcomes, we support maintaining the original 4 TCGA/ProMisE tumour subgroups. We do not consider L1CAM-positive tumours as a distinct EC subtype, but instead as a prognostic subgroup within p53 wt/NSMP tumours and possibly MMR-D tumours. L1CAM expression seems to co-segregate with p53 abn status, and is therefore not a prognostic subgroup within this tumour subtype, and we are unaware of evidence to suggest L1CAM is prognostic within the POLE subtype. Restricting L1CAM IHC use to p53 wt/NSMP (and perhaps the MMR-D) subgroup tumours would provide economic advantage over performing L1CAM IHC on all cases.

L1CAM expression in experimental models has been shown to induce tumour cell migration, invasion, EMT, and chemo resistance.^27,28^ Several of these parameters can plausibly be linked to advanced stage and poor outcomes observed in L1CAM-positive tumours. In this context, a recent study has demonstrated that increased invasiveness of chemoresistant pancreatic cancer cells functionally depends on L1CAM.^35^ It is for these biological reasons that L1CAM is being explored as a therapeutic target in cancer.^17^

The molecular basis for high L1CAM expression in some ECs remains unclear. The p53 wt subgroup comprises a group of EC which, at the time, had no prognostically significant mutation pattern.^16^ However, one mutation observed in up to 50% of this subgroup is activating mutations in *CTNNB1* (encoding β-catenin).^9^ *CTNNB1* mutations occur as part of a mutation module within these carcinomas together with mutations in *KRAS* and *SOX17*, all of which are known to activate Wnt-signalling in colon cancer.^36–38^ Activation of the “canonical” Wnt/β-catenin pathway leads to stabilisation and accumulation of β-catenin within the nucleus, which is thought to induce cell proliferation and EMT.^39–41^ As described above, *L1CAM* is a target gene of β-catenin in colorectal cancer, and its transcription can be induced in EC cell lines by the EMT inducer Slug.^41,42^ We hypothesise that mutations of *CTNNB1* in p53 wt/NSMP subgroup tumours could induce L1CAM expression and thereby promote more aggressive behaviour. *CTNNB1* mutation is concentrated in p53 wt/NSMP tumours and predicts poor outcomes in EC, which raises the possibility that *CTNNB1* mutation analysis or β-catenin IHC could add additional prognostic information to the ProMisE molecular classifier by identifying tumours at higher risk for recurrence within the large p53 wt/NSMP subgroup.^43,44^ Further studies should investigate the correlation between *CTNNB1* mutation and L1CAM expression in p53 wt/NSMP tumours to determine whether these variables are linked and, conclusively, whether to incorporate one or both markers into the ProMisE molecular classifier.

## Conclusion

This study indicates that L1CAM expression status could add important prognostic information to the molecular classification of EC. L1CAM IHC was able to further stratify risk within the p53 wt/NSMP subgroup by identifying carcinomas at higher risk for fatal outcome. A strong correlation between mutation-type p53 immunostaining and L1CAM expression in EC was also confirmed. Taken together with previous studies, our results support adding L1CAM IHC as part of a simplified, clinically applicable molecular classifier for EC. Furthermore, we suggest investigating the role of *CTNNB1* mutation status within the p53 wt/NSMP subgroup of EC and its correlation with aberrant L1CAM expression.

### Data availability statement

The data sets generated during and/or analysed during the current study are available from the corresponding author on reasonable request.

## Electronic supplementary material

Supplementary Figure 1

Supplementary Table 1

## Availability of data and material

The datasets generated during and/or analysed during the current study are available from the corresponding author on reasonable request.
