# Supplementary material for: L1CAM further stratifies endometrial carcinoma patients with no specific molecular risk profile
Source: Br J Cancer. 2018 Jul 27;119(4):480–6. doi: 10.1038/s41416-018-0187-6 (PMC6134076; doi:10.1038/s41416-018-0187-6)

Supplementary Figure 1 L1CAM expression in tumour cells (a) score 0 = 0%, (b) score 1 = 1-10%, (c) score 2 and 3 = ≥ 10%

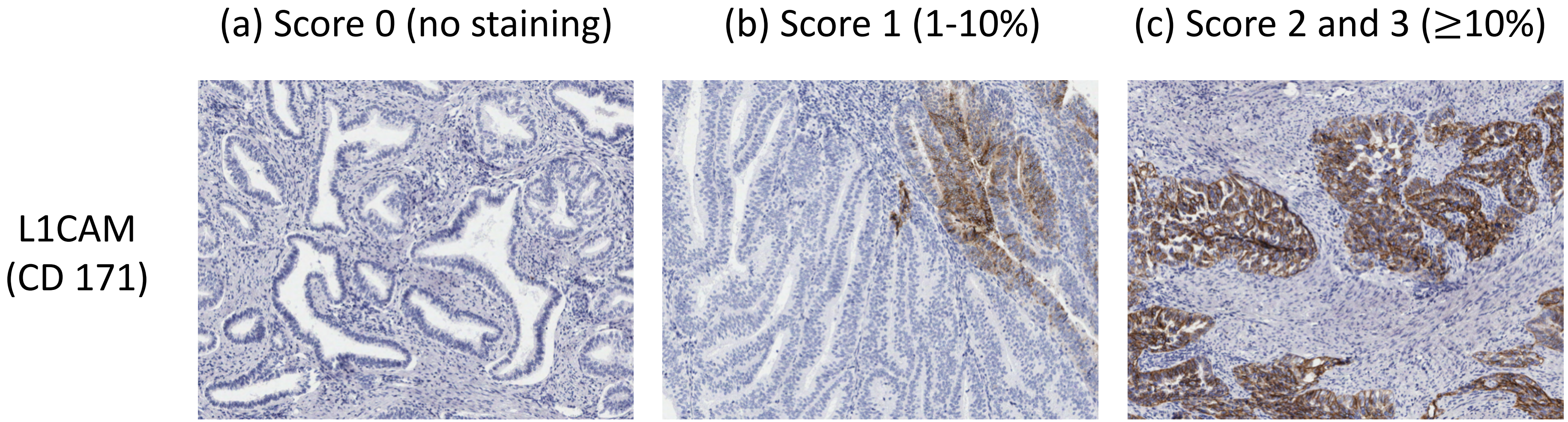

Supplement: Supplementary file 1 — Supplementary Figure 1 [file 41416_2018_187_MOESM1_ESM.pdf]
