# Supplementary material for: L1CAM further stratifies endometrial carcinoma patients with no specific molecular risk profile
Source: Br J Cancer. 2018 Jul 27;119(4):480–6. doi: 10.1038/s41416-018-0187-6 (PMC6134076; doi:10.1038/s41416-018-0187-6)
Supplement: Supplementary file 2 — Supplementary Table 1 [file 41416_2018_187_MOESM2_ESM.pdf]

Supplementary Table 1 Correlation of clinicopathological data and L1CAM in the p53 wt/NSMP ProMisE subgroup.

	Total	L1CAM negative	L1CAM positive	P-Value
**Number of Patients**	228 (100%)	209 (91.6%)	19 (8.4%)	
**Clinicopathological Parameters**				
**Age at diagnosis (yrs)**				
Mean (±sd)	63.2 (±12.4)	62.9 (±12.2)	67.0 (±13.9)	<0.001
Median	63.4	63.0	67.7	
**Stage (FIGO 2009)**				
I	198 (86.8%)	185 (88.5%)	13 (68.4%)	0.03
II-IV	30 (13.2%)	24 (11.5%)	6 (31.6%)	
**Tumour grade**				
Grade 1 and 2	211 (92.5%)	198 (94.7%)	13 (68.4%)	<0.001
Grade 3	17 (7.5%)	11 (5.3%)	6 (31.6%)	
**LVSI**				
negative	213 (93.4%)	196 (93.7%)	17 (89.5%)	0.4
positive	15 (6.6%)	13 (6.3%)	2 (10.5%)	
**Histology**				
Endometrioid	226 (99.1%)	208 (99.5%)	18 (94.7%)	0.5
Non-Endometrioid	2 (0.9%)	1 (0.5%)	1 (5.3%)	
